# Acute and Subacute Oral Toxicity Assessment of The Polysaccharides Extracted from *Clinacanthus nutans* Leaves: A Preclinical Model for Drug Safety Screening

**DOI:** 10.21315/tlsr2025.36.1.13

**Published:** 2025-03-30

**Authors:** Tan Yong Chia, Chee-Yuen Gan, Gurjeet Kaur, Pike-See Cheah, Vikneswaran Murugaiyah, Ashfaq Ahmad, Bader Alsuwayt, Sulaiman Mohammed Abdullah Alnasser, Muhammad Hakimin Shafie, Selvamani Narayan Nair, Mohammed H Abdulla, Edward James Johns

**Affiliations:** 1 Analytical Biochemistry Research Centre (ABrC), Universiti Innovation Incubator Building, SAINS@USM Campus, Universiti Sains Malaysia, Lebuh Bukit Jambul 11900 Pulau Pinang, Malaysia; 2 Department of Clinical Medicine, Advanced Medical and Dental Institute, Universiti Sains Malaysia, Bertam 13200 Kepala Batas, Pulau Pinang, Malaysia; 3 Institute for Research in Molecular Medicine (INFORMM), Universiti Sains Malaysia, 11800 USM Pulau Pinang, Malaysia; 4 Department of Human Anatomy, Faculty of Medicine and Health Sciences, Universiti Putra Malaysia, 43000 Serdang, Selangor, Malaysia; 5 Department of Pharmacology, School of Pharmaceutical Sciences, Universiti Sains Malaysia, 11800 USM Pulau Pinang, Malaysia; 6 Centre for Drug Research, Universiti Sains Malaysia, 11800 USM Pulau Pinang, Malaysia; 7 Department of Pharmacy Practice, College of Pharmacy, University of Hafr Al Batin, Al Jamiah, Hafar Al Batin 39524, Saudi Arabia; 8 Department of Pharmacy, Qassim University, Buraydah 52571, Saudi Arabia; 9 Department of Physiology, School of Medicine, University College of Cork, T12 K8AF Cork, Ireland

**Keywords:** *Clinacanthus nutans*, Biochemistry, Haematology, Polysaccharides, Toxicology, *Clinacanthus nutans*, Biokimia, Hematologi, Polisakarida, Toksikologi

## Abstract

Emerging investigations have indicated that many plant polysaccharides may be beneficial for treating metabolic diseases. To date, the therapeutic efficacy and potential toxicity of polysaccharides extracted from *Clinacanthus nutans* (*C. nutans*) remain unexplored. This study investigated the in vivo acute and subacute oral toxicological profiles of the highest doses of *C. nutans* bioactive polysaccharides (CNBP) extracted from the leaves using conventional toxicity methods. The total of 39 healthy 8–10 weeks male Sprague-Dawley rats (*n* = 3) were randomly assigned to control (C), acute (A) and subacute (SA) groups receiving 125, 250, 500, 1,000, 2,000 or 3,000 mg/kg/day of CNBP extract, respectively. The acute group received a single dose of CNBP extract, whereas the subacute group received daily single doses of CNBP extract for 14 days. Oral administration of up to 3,000 mg/kg CNBP extract caused no abnormal signs of toxicity during 14 days. However, daily administration of 500 mg/kg or higher doses of CNBP extract for 14 days induced a mild degree of toxicity in the liver, characterised by elevated alkaline phosphatase levels with C (163 ± 9 U/L) vs. SA500 (222 ± 49 U/L), SA1000 (223 ± 29 U/L), SA2000 (238 ± 33 U/L) and SA3000 (252 ± 18 U/L). CNBP extracts exhibit therapeutic potential, exemplified by diuretic, natriuretic, anti-hypertensive, anti-tachycardia, reno-protective and cholesterol-lowering properties. Precautions should be taken when administering the extracts at higher doses and for longer durations.

## Abstract (fr)

**Abstrak:**

Penyiasatan baru menunjukkan bahawa kebanyakan polisakarida tumbuhan mungkin bermanfaat untuk merawat penyakit metabolik. Setakat ini, keberkesanan terapeutik dan potensi ketoksikan polisakarida yang diekstrak daripada *Clinacanthus nutans* (*C. nutans*) masih belum diterokai. Kajian ini menyiasat profil toksikologi oral akut dan subakut in vivo bagi dos tertinggi *C. nutans* dengan menggunakan polisakarida bioaktif (CNBP) yang diekstrak daripada daun dengan kaedah ketoksikan konvensional. Sebanyak 39 ekor tikus Sprague-Dawley jantan berumur 8–10 minggu yang sihat (*n* = 3) dibahagikan secara rawak dan ditugaskan sebagai kumpulan mengawal (C), akut (A) dan kumpulan sub-akut (SA) masing-masing menerima 125, 250, 500, 1,000, 2,000 atau 3,000 mg/kg/hari ekstrak. Kumpulan akut menerima satu dos ekstrak CNBP, manakala kumpulan sub-akut menerima dos tunggal ekstrak CNBP setiap hari selama 14 hari. Pemberian oral sehingga 3,000 mg/kg ekstrak CNBP tidak menyebabkan tanda-tanda ketoksikan yang tidak normal dalam tempoh 14 hari. Manakala, pemberian harian 500 mg/kg atau dos ekstrak CNBP yang lebih tinggi selama 14 hari menyebabkan tahap ketoksikan ringan dalam hati, yang dicirikan oleh paras fosfatase alkali yang tinggi dengan C (163 ± 9 U/L) berbanding dengan SA500 (222 ± 49 U/L), SA1000 (290 ± 2), SA1000 (290 ± 0,000), SA1000. 33 U/L) dan SA3000 (252 ± 18 U/L). Ekstrak CNBP mempamerkan potensi terapeutik, dicontohkan oleh sifat diuretik, natriuretik, anti-hipertensi, anti-takikardia, pelindung reno dan penurun kolesterol. Langkah berjaga-jaga perlu diambil semasa mengendalikan ekstrak pada dos yang lebih tinggi dan untuk tempoh yang lebih lama.

**Highlights**

- *Clinacanthus nutans* bioactive polysaccharides (CNBP) extracts exhibit therapeutic potential, exemplified by diuretic, natriuretic, anti-hypertensive, anti-tachycardia, reno-protective and cholesterol-lowering properties. However, precautions should be taken when administering the extracts at higher doses and for longer durations.
- Oral administration of a single dose of CNBP extract (up to 3,000 mg/kg) caused no abnormal signs of toxicity on the entire 14 days study period.
- Daily administration of 500 mg/kg or higher doses of CNBP extract for 14 days induced a mild degree of toxicity in the liver, characterised by elevated alkaline phosphatase levels with C (163 ± 9 U/L) vs. SA500 (222 ± 49 U/L), SA1000 (223 ± 29 U/L), SA2000 (238 ± 33 U/L) and SA3000 (252 ± 18 U/L).

## INTRODUCTION

Phytomedicine, also known as phytotherapy, is a widely used modality in complementary and alternative medicine. International surveys report that a significant proportion of the world’s population relies on traditional herbal medicine for their healthcare needs (Nsagha *et al*. 2020). In addition, the general perception of many is that herbal remedies are safe and devoid of harmful effects. However, it is well known that there may be an association between phytomedicine and potential adverse effects such as organ damage and even life-threatening conditions (Staines 2011; Posadzki *et al*. 2013; Başaran *et al*. 2022). Therefore, toxicological screening of ethnobotanical compounds and their potential pharmacological efficacy could reveal risks associated with the use of herbal remedies.

*Clinacanthus nutans* and *C. nutans* (Burm. f) Lindau, which is affiliated to the Acanthaceae family, has recently attracted the attention of researchers from sub-tropical Asian countries, including Malaysia, Brunei and Singapore, owing to its abundant active secondary metabolites that exhibit certain pharmacological effects in humans, such as anti-diabetic, anti-hypertensive, anti-inflammatory and antioxidant properties. Other pharmacological activities such as anti-venom, anti-cancer, anti-bacterial, anti-fungal as well as analgesic activities have also been reported (Chia *et al*. 2021a).

Locally, *C. nutans* extracts are prepared using a decoction technique, in which fresh leaves are boiled with water and consumed as herbal tea. However, in phytochemical investigations, the extractions are executed with organic solvents such as ethanol, methanol, hexane and petroleum ether (Haida & Hakiman 2019). Several bioactive elements, including phenolic compounds, sulphur-containing compounds, glycosides, terpene-tripenoids and terpene-phytosterols, have been identified as possessing therapeutic properties. However, paradoxically it also has some reported toxic effects at certain dose levels as evident in in-vivo experiments (Chia *et al*. 2021a).

Polysaccharides are nontoxic, naturally biodegradable biopolymers formed from biomacromolecules that occur widely in nature. These polysaccharides consist of 10 or more simple sugar molecules, known as monosaccharides, which are connected by glycosidic linkages and can vary considerably in size, structural complexity and sugar content. They can be linear or highly branched, composed of homopolysaccharides or heteropolysaccharides generated from monosaccharide units that confer distinct physical and chemical properties (Benalaya *et al*. 2024). These compounds have wide-ranging functions such as anti-coagulation (Wang *et al*. 2020); corneal endothelium protection, human joint lubrication, skin moisturisation (Liao *et al*. 2005; Vasvani *et al*. 2020), and in the treatment of diabetes mellitus (Meneguin *et al*. 2021). However, rigorous scientific investigations related to isolation, purification and structural characterisation of bioactive polysaccharides from *C. nutans* are still rare. Therefore, the objective of the current study was to evaluate the potential acute and subacute oral toxicity of polysaccharides extracted from *C. nutans* leaves and to determine their corresponding pharmacological actions for use in future disease prevention strategies.

## MATERIALS AND METHODS

### Plant Procurement and Botanical Identification

Fresh leaves of *C. nutans* were collected from Foong Lee Plantation, Kampung Baharu Pondok Tanjong, 34010 Taiping, Perak, Malaysia with (GPS Coordinate: 5.008603839672635, 100.73065334028212). The botanical authentication of the plant specimens comprising flowers, leaves and roots was confirmed at the Unit Herbarium, School of Biology, Universiti Sains Malaysia, and voucher number 11153 from the specimen was deposited in the herbarium for future reference.

### Leaves Preparation

The fresh leaves of *C. nutans* were carefully separated from the stems and the leaves were washed with distilled water and dried overnight in a well-ventilated room. The leaves were further lyophilized at −40°C and milled into powder. The obtained powders were sieved using (60-mesh screen) and stored at 4°C until use. All chemicals (citric acid and ethanol) used in this experiment were of analytical grade and acquired from Sigma-Aldrich, Malaysia (Shafie *et al*. 2019).

### Crude Polysaccharides Extraction

The crude polysaccharides were extracted by adapting previously published methods with slight modifications and were executed using a conical flask with an incubator shaker (IKA KS 4000i, Germany). Briefly, 5 g of lyophilised *C. nutans* powder was added to 125 mL of 0.1M citrate-phosphate buffer at pH 2 with a solid-to-buffer ratio of 1:25; these homogenates were then placed in an incubator shaker (IKA KS-4000-i Control, Staufen, Germany) and constantly shaken at 250 rpm for 120 min at 80°C. The slurry was then filtered with an ultrafine mesh density muslin cloth (pore size, 50 μm–75 μm) in two layers. Subsequently, the filtrates were centrifuged at 5,000 rpm for 20 min at 20°C to remove the remaining small-molecular-weight molecules from the extract. The filtrates were then isolated with four volumes of 99% (v/v) ethanol at a ratio of (1:4) and precipitated overnight in a freezer at 4°C. Following this, the supernatant layer was carefully decanted and the resultant *C. nutans* bioactive polysaccharide (CNBP) was then lyophilized and ground into powder. CNBP was stored in a desiccator until further use (Shafie *et al*. 2019; Kamarudin & Gan 2016; Tan & Gan 2016).

### Preparation of Experimental Animals

Thirty-nine healthy male Sprague-Dawley rats, weighing 200 g–250 g between 8–10 weeks of age, were obtained from the Animal Research Unit of the Advanced Medical and Dental Institute, Universiti Sains Malaysia. All experimental procedures and protocols were conducted following the approval of the Animal Research and Service Centre (ARASC) of Universiti Sains Malaysia with approval code: USM/IACUC/2021/(131)(1162), and the study was conducted in accordance with the basic and clinical pharmacology and toxicology policy for experimental and clinical studies (Sarega *et al*. 2016b). Animals were housed in a standard animal facility (temperature, 24°C, humidity, 60%–70%) with a 12h:12h day light-dark cycle and housed individually in cages provided by the Centre of Drug Research, Universiti Sains Malaysia. The animals were randomly assigned, marked to permit individual identification, and kept in their cages for at least five days prior to dosing to allow for acclimatisation to the laboratory conditions and any non-specific stress. Rats were allowed free access to chow and filtered tap water *ad libitum* prior to the beginning of the toxicity study. Investigations were carried out according to the instructions of the Organisation for Economic Cooperation and Development Guidelines (OECD-GL) for Acute Oral Toxicity test-GL423 and the subacute oral toxicity test-GL407 with slight modifications (Aliyu *et al*. 2020; Organisation for Economic Co-operation and Development [OECD] 2001; 2008).

### Acute and Subacute Oral Toxicity Study

Three animals (*n* = 3 per group) were used for each investigated dose level. CNBP suspension formulations were prepared in a graded manner, adjusted for individual body weight, by mixing CNBP powder with distilled water to produce a suspension.

In the acute oral toxicity protocols, each animal was administered a single dose of the CNBP suspension via an intragastric catheter. The CNBP suspension was administered once after the animals were fasted for 18 h, but allowed water *ad libitum*. One control group (C) received only filtered tap water, while the other six groups received only a single dose of CNBP suspension as follows: (A125) received 125 mg/kg, (A250) received 250 mg/kg, (A500) received 500 mg/kg, (A1000) received 1,000 mg/kg, (A2000) received 2,000 mg/kg and (A3000) received 3,000 mg/kg. Animals were then observed for symptoms of acute toxicity, that is, mortality and behavioural changes including aggression, agitation, asphyxia, ataxia, catatonia, convulsion, fasciculation, prostration, sedation and somnolence, tremor, unusual vocalisation and unusual locomotion for the first 30 min after the first hour, followed hourly over the subsequent 8 h, and then periodically up to 48 h. Daily general behaviour, body weight changes, morbidity signs and mortality were observed continuously until the end of day 14 of the study period (OECD 2001).

The subacute toxicity protocol comprised repeated daily oral doses of CNBP for 14 days. The rats were randomly distributed into six groups to receive the same doses each day, as in the acute toxicity protocol. The CNBP suspensions were orally administered daily throughout the 14-day study period at doses of 125 mg/kg (SA125), 250 mg/kg (SA250), 500 mg/kg (SA500), 1,000 mg/kg (SA1000), 2,000 mg/kg (SA2000) or 3,000 mg/kg (SA3000). Along with water and food consumption, signs of toxicity, as noted in the acute toxicity experiment, were recorded per diem over the whole 14-day cycle (OECD 2008).

### Physiological Data Measurements

Body weight, water intake, urine output and food intake parameters were recorded on days 0, 7 and 14. Body weight was measured using an electronic digital balance (Letica LE 2066, Scientific Instrument, Barcelona, Spain) before the commencement of the first oral administration of the CNBP suspension. Water intake, urine output and food intake parameters were measured using metabolic cages (Nalgene^®^, Thermo Scientific, Philadelphia, USA). Animals were placed in metabolic cages, where they were kept for 24 h. The water and food intake for each animal was measured by subtracting the amount remaining in the graded feeding bottle from the amount measured initially. At the end of 24 h, the amount of urine collected was recorded as the urine output volume. Urine samples were stored in a disposable test tube (FC Bios, Sdn. Bhd., Malaysia), centrifuged at 3,000 rpm for 10 min (Hettich EBA 8S, Zentrifugen, Hettich Instruments USA) to remove impurities, and stored at −30°C in a freezer (Sanyo Electric Co., Ltd., Japan) to be used later in biochemical analyses (Liaskou *et al*. 2012). The fractional sodium excretion (FENa^+^) and creatinine clearance (CrCl) were calculated using standard equations as previously reported (Chia *et al*. 2021b).

### Non-invasive Blood Pressure Measurements

The weekly systolic blood pressure (SBP), diastolic blood pressure (DBP), mean arterial blood pressure (MAP), and heart rate (HR) were measured in conscious rats using CODA^®^ tail cuff plethysmography (Kent Scientific Corporation, Torrington, CT, USA). At each session, 20 consecutive readings were measured from each rat, and average readings were calculated (Chia *et al*. 2020).

### Blood and Organ Sampling

On Day 15, all animals were fasted overnight and sacrificed using an overdose of 100 mg/kg sodium pentobarbitone (Nembutal^®^, CEVA, Santé Animale, Libourne, France). Blood samples were collected via cardiac puncture into an ethylenediaminetetraacetic acid (EDTA)-coated vacutainer tube (BD Vacutainer^®^, Becton, Dickinson & Co., USA) for haematological investigations. The livers and kidneys were collected for necropsy (Chia *et al*. 2020; 2021a; 2021b).

### Haematological and Biochemical Parameters

Blood samples were analysed for haemoglobin concentration, red blood cell (RBC) count, packed cell volume (PCV), mean corpuscular volume (MCV), mean corpuscular haemoglobin (MCH), mean corpuscular haemoglobin concentration (MCHC), red cell distribution width (RDW), white blood cell (WBC) count, lymphocytes, monocytes, neutrophils, basophils, eosinophils and platelet count.

Renal function parameters were measured, including urine sodium, plasma sodium, urine potassium, plasma potassium, urine creatinine, plasma creatinine, plasma urea, plasma chloride and fasting blood glucose index. Liver function tests were performed to measure total protein, albumin, globulin, albumin/globulin ratio, total bilirubin, alkaline phosphatase, gamma-glutamyl transferase (GGT), aspartate aminotransferase (AST) and alanine aminotransferase (ALT) levels. The blood lipid profile was examined and included cholesterol, high-density lipoprotein (HDL), low-density lipoprotein (LDL), non-high-density lipoprotein cholesterol, triglycerides and the total cholesterol/high-density lipoprotein ratio (Farsi *et al*. 2016).

### Organ Weight and Histopathological Evaluation

After sacrificing the animals, the livers and kidneys were harvested and rinsed with normal saline to wash off excess blood. Both organs were blotted dry using filter paper (Whatman^®^ cellulose filter paper, Merck, Germany), after which the organs were weighed. The organs were then fixed in 10% neutral buffered formalin. The organs were embedded in paraffin and sectioned into 5μm slices using a microtome (Accu-Cut, Sakura Finetek, USA), followed by stepwise dehydration using alcohol-xylene solvents and staining with haematoxylin-eosin. Tissue slides were examined for histopathological degeneration with respect to those of control animals. The relative organ weight (ROW) of each organ was calculated using the following equation (Farsi *et al*. 2016):


$$\text{ROW}=\frac{\text{Absolute organ weight}}{\text{Fasted body weight on sacrifice day}}\times 100\%$$

### Statistical Analysis

Statistical analysis was performed using GraphPad Prism^®^ Version 9.0 software (GraphPad Software, San Diego, California, USA). All data are expressed as mean ± SEM, and significant differences were accepted as (*P* ≤ 0.05). Data from physiological and non-invasive blood pressure measurements were analysed using repeated measures Analysis of Variance (ANOVA). Other haematological and biochemical data were analysed using one-way ANOVA followed by the Bonferroni post hoc test.

## RESULTS

### No-Observed-Adverse-Effect Level (NOAEL) Limit Test

Intragastric administration of CNBP at doses of 125 mg/kg, 250 mg/kg, 500 mg/kg, 1,000 mg/kg, 2,000 mg/kg and 3,000 mg/kg did not produce any clinical signs of morbidity- or toxicity-related symptoms in either the acute or subacute animals during the experimental period. All the animals survived until the end of the observation period. Likewise, no gross anatomical abnormalities were observed in the organs during autopsy. This result indicated that the LD_50_ was above 3,000 mg/kg for the CNBP extracts.

### Effect of CNBP Extracts on Physiological Parameters

Data for body weight, water intake, urine output and food intake for both the acute and subacute models are shown in Table 1. Significant (*P* ≤ 0.05) progressive body weight gain was observed in all experimental animals during the study period. Oral administration of CNBP extracts at all doses did not result in significant changes in water or food intake. However, in animals in groups A2000 and A3000, there was a significant (*P* ≤ 0.05) increase in urine output volume on day 14. Similar findings were observed in SA1000, SA2000 and SA3000 animals.

### Effect of CNBP Extracts on Hemodynamic Parameters

SBP, DBP, MAP and HR findings are presented in Table 2. The SBP in the control and animals remained stable throughout the study period. However, at the end of day 14, the SBP in the A2000 and A3000 acute study animals was significantly (*P* ≤ 0.05) lower than that in the control animals. Furthermore, subacute administration of CNBP extracts did not affect the SBP of the subacute groups SA125 and SA250 over the entire study period; however, in the SA500, SA1000, SA2000 and SA3000 groups, SBP was significantly (*P* ≤ 0.05) lower than that in the control group. The DBP of the SA125, SA250 and SA500 subacute groups did not change, but DBP in the SA1000, SA2000 and SA3000 groups was significantly (*P* ≤ 0.05) lower on day 14 compared to day 0 baseline. Likewise, the MAP of the groups subjected to 1,000 mg/kg, 2,000 mg/kg and 3,000 mg/kg of CNBP extract in both acute and subacute groups was significantly (*P* ≤ 0.05) decreased on day 14. A similar pattern was also observed in HR, as in the A2000, A3000, SA2000 and SA3000 groups, HR was significantly (*P* ≤ 0.05) lower from day 7 to day 14. On the final day of the study, the HR of both acute and subacute groups treated with 1,000 mg/kg, 2,000 mg/kg and 3,000 mg/kg of CNBP extracts was significantly (*P* ≤ 0.05) reduced compared to the control group.

### Effect of CNBP Extracts on Haematological and Biochemical Parameters

The haematological parameters, haemoglobin, RBC, PCV, MCV, MCH, MCHC and RDW for both acute and subacute SD rats treated with CNBP extracts are tabulated in Table 3. The results showed that oral administration of CNBP extracts at all doses from day 1 to day 14 did not significantly change haemoglobin, RBC, PCV, MCV, MCH, MCHC or RDW in either the acute or subacute groups compared to the control group. Similarly, no significant differences in WBC parameters, such as lymphocytes, monocytes, neutrophils, eosinophils, basophils and platelets, were observed in the control or CNBP-treated animals (Table 4).

Table 5 shows that there was a significant (*P* ≤ 0.05) increase in the urinary sodium concentration and fractional excretion of sodium in the A500, A1000, A2000 and A3000 groups. A similar increase in urinary sodium concentration was observed in the SA250, SA500, SA1000, SA2000 and SA3000 subacute groups on day 14 when compared to the control group (Table 5). The same pattern of observations was also found in the renal functional parameters; the urinary creatinine concentration and creatinine clearance were significantly (*P* ≤ 0.05) higher in the A1000, A2000 and A3000 groups than in the control group. Furthermore, treatment with CNBP extracts significantly (*P* ≤ 0.05) enhanced the urinary creatinine concentration and creatinine clearance in the SA250, SA500, SA1000, SA2000 and SA3000 groups compared to the control group on day 14 (Table 6).

Most liver functional parameters were minimally affected by CNBP extract administration in all the acute treatment groups (Table 7). Table 8 demonstrates that oral administration of CNBP extracts significantly (*P* ≤ 0.05) elevated alkaline phosphatase levels in SA500, SA1000, SA2000 and SA3000 animals. CNBP extracts administered to the A500, A1000, A2000 and A3000 groups had significantly (*P* ≤ 0.05) lower non-HDL levels than control animals (Table 9). Moreover, administration of CNBP extracts significantly (*P* ≤ 0.05) reduced both LDL and non-HDL levels in the subacute groups. The CNBP extracts did not cause any significant effects at any dose on other lipid profiles, that is, total cholesterol, HDL, triglycerides and total cholesterol/HDL ratio (Table 9).

### Evaluation of CNBP Extracts on Organ Weight and Histopathology

Administration of CNBP extract had no significant effect on the relative organ weights of the liver and kidneys in either the acute or subacute groups after 14 days (Table 10).

**Figure 1:**
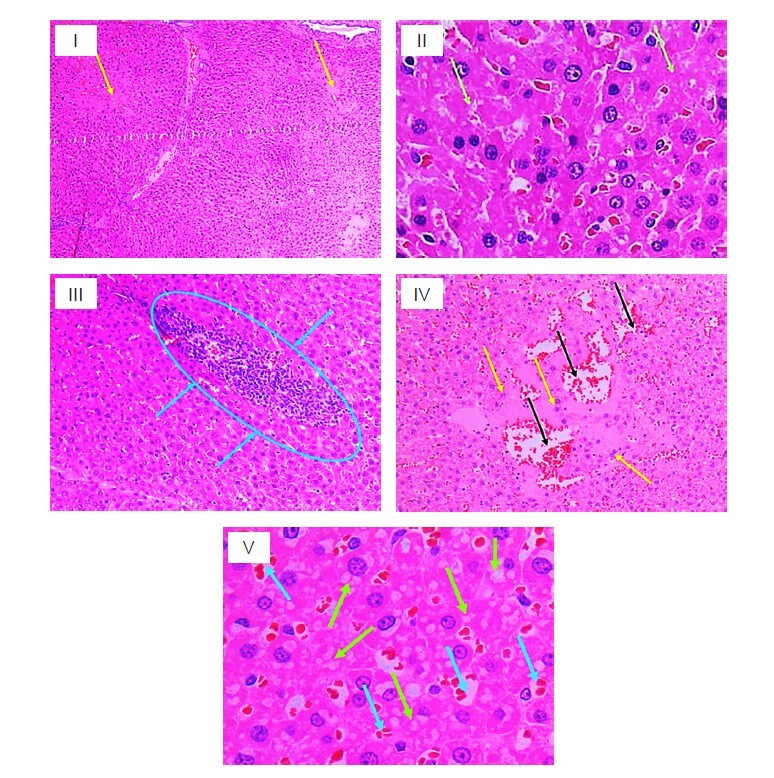
Photomicrographs of SD rats’ liver. (I) Hepatocyte necrosis with eosinophilic cytoplasm, karyolysis and degeneration of hepatocytes (yellow arrows) – magnification ×40; (II) Necrotic hepatocytes (green arrows) – magnification ×400; (III) Portal inflammation – magnification ×100; (IV) Hepatocyte necrosis (yellow arrows), congestion and dilatation of veins (black arrow) – magnification ×100; and (V) Hydropic degeneration and necrotic hepatocytes (green arrows), and sinusoidal congestion (blue arrow) – magnification ×400.

**Figure 2:**
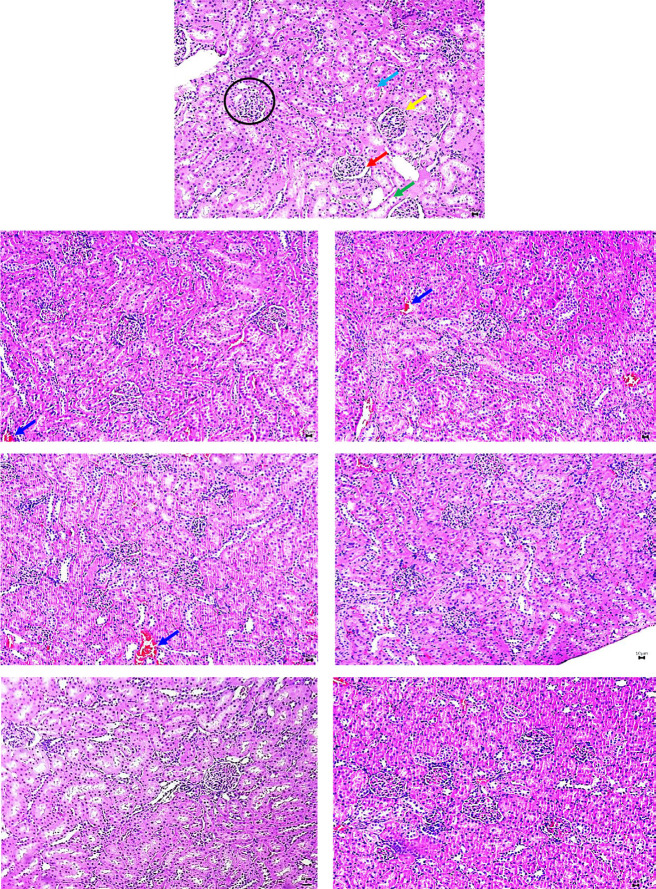
Photomicrographs of SD rats kidney section with acute study; C, A125, A250, A500, A1000, A2000 and A3000 groups treated with CNBP. (Sections were stained with H&E; 10×). Samples revealed normal renal tissue histology at all dosages administrated via acute with normal renal glomeruli and tubular system. There was no evidence of glomerular changes, degeneration and necrosis of renal tubular epithelial cells or significant interstitial inflammation. The photomicrographs depict the glomerulus (black circle), Bowmen’s capsule (yellow arrow), Bowmen’s space (red arrow), proximal tubule (light blue arrow), distal tubule (light green arrow) and blood capillaries (blue arrow).

**Figure 3:**
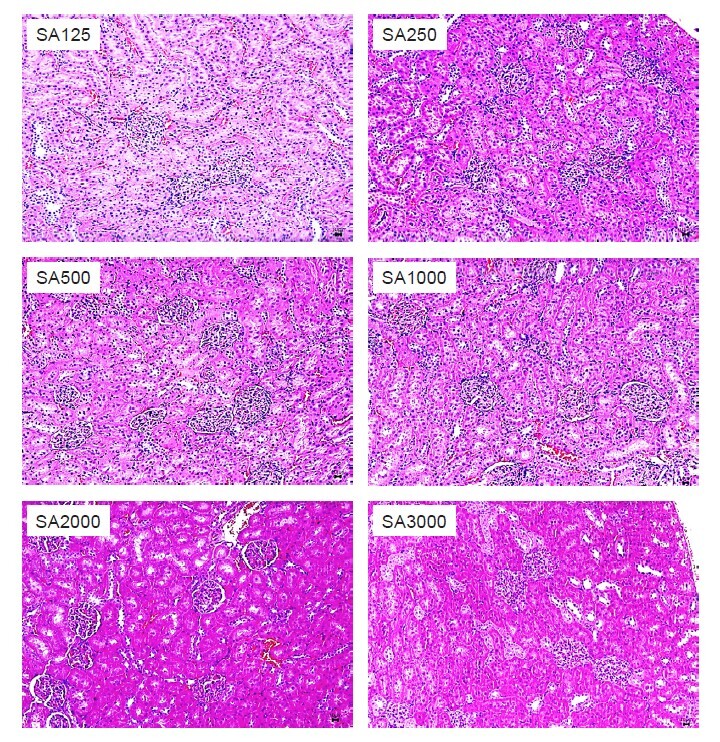
Photomicrographs of SD rats kidney section with subacute study; SA125, SA250, SA500, SA1000, SA2000 and SA3000 groups treated with CNBP. (Sections were stained with H&E; 10×). Samples have demonstrated normal renal glomeruli and tubular system at all dosages administrated via subacute manners. No evidence of glomerular changes, degeneration and necrosis of renal tubular epithelial cells or significant interstitial inflammation was seen.

The light microscopic examination of liver and kidney sections of all experimental groups are presented in Figs. 1, 2 and 3. H&E-stained liver samples revealed a normal histological appearance in most of the treated groups. Immune cell infiltration was not observed in tissue sections. Mild vascular (sinusoidal and venous) congestion was observed in some samples from SA500, SA1000, SA2000 and SA3000 animals administered the acute and subacute protocols (Fig. 1 I–V). In Photomicrographs of SD rats’ liver section with acute study; C, A125, A250, A500, A1000, A2000 and A3000 groups treated with CNBP. Mild to moderate toxicity effects were detected in the groups administered 500 mg/kg, 1,000 mg/kg, 2,000 mg/kg and 3,000 mg/kg extract. Consisted of mild to moderate hydropic degeneration, cytoplasmic vacuolations, eosinophilic cytoplasm of hepatocytes, necrosis, activated Kupffer cells, sinusoidal and venous congestion/dilatation and mild portal tract inflammation. These changes were moderate to severe in the group administered 1,000 mg/kg extract.

On the other hand, in Photomicrographs of SD rats’ liver section with subacute study; SA125, SA250, SA500, SA1000, SA2000 and SA3000 groups treated with CNBP. Mild vascular (sinusoidal and venous) congestion was observed in some samples from the 500 mg/kg, 1,000 mg/kg, 2,000 mg/kg and 3,000 mg/kg groups administered via subacute manner.

The effects of CNBP extracts on the H&E-stained kidney samples revealed a normal histology of renal glomeruli and tubular system at all dosages administered in the acute and subacute groups (Figs. 2 and 3). There was no evidence of glomerular changes, degeneration, necrosis of renal tubular epithelial cells or significant interstitial inflammation.

## DISCUSSION

The present study was conducted to determine the toxicity potential of CNBP extracts by evaluating their safety profile over 14 days. This was done by measuring physical signs and haematological, biochemical, and histopathological evaluations of vital organs in both acute and subacute studies in rats. Data obtained from both the acute and subacute toxicity studies showed that a single dose or repeated daily doses of CNBP extract for 14 days resulted in no morbidity, mortality, or changes in the general appearance, eye and skin colour. In addition, the extract had no effect on appetite or growth. Additionally, physiological data revealed a significant increase in urinary volume excretion and fractional sodium excretion in acute experimental animals treated with 2,000 mg/kg and 3,000 mg/kg of CNBP extract. Similar observations were also observed in subacute experimental animals treated with 1,000 mg/kg, 2,000 mg/kg and 3,000 mg/kg CNBP extracts. The results also demonstrated that oral administration of CNBP extracts enhanced diuretic activity in the kidney in agreement with previous studies in the literature (Ismail & Ilya 2017; Widjaja & Ichwan 2021).

The CNBP extract in this study produced a dose-dependent decrease in systolic, diastolic and mean blood pressure, as well as in heart rate. Systolic blood pressure was lower on day 14 in both the acute and subacute groups than in their control counterparts. Systolic blood pressure decreased in acute groups receiving 2,000 mg/kg and 3,000 mg/kg and in the subacute groups receiving 500 mg/kg to 3,000 mg/kg of CNBP extract. The heart rate decreased in both acute and subacute groups treated with 1,000 mg/kg, 2,000 mg/kg and 3,000 mg/kg of CNBP extract compared to their control counterparts. This reduction in blood pressure and heart rate is consistent with the perceived traditional use of *C. nutans* as an antihypertensive agent (Chia *et al*. 2021a; Haida & Hakiman 2019). A similar observation was also reported by Sarega *et al*. (2016b), who studied the effects of phenolic-rich extracts of *C. nutans*. The study showed that the antihypertensive effect of *C. nutans* extracts could be due to the high content of K^+^ in the extract compared to Na^+^, which reflects a very low Na^+^/K^+^ ratio. This would be favourable from a nutritional point of view, as diets with a low Na^+^/K^+^ ratio are associated with lower incidence of hypertension (Sarega *et al*. 2016a). This argument was further supported by the fact that activation of the dose-dependent hypotensive, bradycardic and vasorelaxant effects of *C. nutans* could possibly be mediated through a nitric oxide-dependent mechanism (Nwokocha *et al*. 2021).

The kidney functions are known to be influenced by drugs and phytochemicals of plant origin that may ultimately lead to renal insufficiency (Debelo *et al*. 2015). Assessment of possible renal damage due to CNBP extracts in the present study was made by analysing plasma and urine concentrations of creatinine as well as sodium and potassium. In most of the treatment groups, there were no significant alterations in plasma creatinine levels. However, there was a significant increase in creatinine clearance in animals receiving a single dose of 1,000 mg/kg, 2,000 mg/kg and 3,000 mg/kg of CNBP extracts or repeated doses of 250 mg/kg, 500 mg/kg, 1,000 mg/kg, 2,000 mg/kg and 3,000 mg/kg of CNBP extracts. Furthermore, fractional sodium excretion was also enhanced in both the acute and subacute study groups at doses of 250 mg/kg and 500 mg/kg, respectively. The administration of CNBP extracts did not cause nephrotoxicity, as supported by histopathological analysis and microscopic examination of the kidneys which showed a normal cell architecture. Additionally, there was no evidence of glomerular changes, interstitial inflammation, degeneration or necrosis of renal tubular epithelial cells.

The liver, via the hepatic portal vein, is the primary organ exposed to ingested nutrients and toxic substances (Debelo *et al*. 2015; Liaskou *et al*. 2012). Consequently, elevated plasma levels of enzymes produced by the liver indicate insult or damage. In the present study, there were no significant changes in the levels of total protein, albumin, globulin, total bilirubin, GGT, AST and ALT in either the acute or subacute groups at any of the tested doses. This implies that CNBP extract had minimal effects on the liver. This view was further supported by histopathological examination of the liver, which showed normal architecture in all groups and treatments. However, a significant elevation in ALP level was noted in the subacute groups administered 500 mg/kg, 1,000 mg/kg, 2,000 mg/kg and 3,000 mg/kg of CNBP extract. This observation was consistent with the histopathological study which showed mild vascular (sinusoidal and venous) congestion (Fig. 1). Nevertheless, these significant changes were within the normal laboratory range and did not necessarily indicate hepatotoxicity (Farsi *et al*. 2016). There were no significant changes in the relative organ weights of the pairs of kidneys or the liver in any of the experimental groups. Macroscopic examination of the kidneys and livers of the various groups receiving CNBP extract showed no difference in colour compared to the control groups. These macroscopic and microscopic findings in the kidneys and liver would reinforce the view of the non-toxic nature of *C. nutans* extract. In contrast, other studies have reported abnormal histopathological changes to be present when using ethanolic *C. nutans* extracts, appearing as centrilobular sinusoid dilatation/centrilobular necrosis, hydropic degeneration/cytoplasmic vacuolation, and inflammation in liver tissues in medium- and high-dose groups (Asyura *et al*. 2016). Therefore, circumspection should be taken when administering the extracts at higher doses and longer durations. Based on the current results, a single dose or continuous administration of 3,000 mg/kg for 14 days would be considered safe according to OECD guidelines. Therefore, the LD_50_ of CNBP extract was predicted to be greater than 3,000 mg/kg/day.

Cholesterol is an essential lipophilic molecule required for normal cell functioning and is a precursor molecule in the synthesis of steroid hormones. Dysregulation of cholesterol metabolism is a risk factor involved in the aetiology of several chronic diseases such as cardiovascular diseases (Başaran *et al*. 2022; Sarega *et al*. 2016a). In the present investigation, plasma LDL and non-HDL were significantly lower in the acute group treated with 500 mg/kg, 1,000 mg/kg, 2,000 mg/kg and 3,000 mg/kg of CNBP extract and in the subacute group animals receiving 250 mg/kg, 500 mg/kg, 1,000 mg/kg, 2,000 mg/kg and 3,000 mg/kg of CNBP extract. This points to the suggested anti-hyperlipidaemic effects of *C. nutans* extract, in agreement with a previous study (Sarega *et al*. 2016b). Although *the C. nutans* extract used in the present investigation comprised plant polysaccharides, administration of the CNBP extract did not induce any significant fluctuation in the fasting blood glucose index in any group. The findings from the present investigation are in agreement with the report that flavonoids compounds from *C. nutans* attenuate atherosclerosis progression in rats with type 2 diabetes by reducing vascular oxidative stress and inflammation (Azemi *et al*. 2021). It has also been reported that *C. nutans* is richly endowed with several phenolic compounds which have therapeutic potential against diabetic vascular diseases and can improve kidney histopathological features in the diabetic rat model (Azemi *et al*. 2021; Mas’ ulun *et al*. 2021). The findings of the present investigation demonstrated that the CNBP extract did not have any significant impact on erythrocyte indices in any of the groups treated. Hence, acute and subacute treatment with CNBP extracts had no effect on the size of RBCs or haemoglobin weight per RBC in rats. This observation clearly suggests that the extracts did not induce macrocytic and microcytic anaemia or jaundice over 14 days of administration. The findings of the present investigation of minimal haematological effects were consistent with other reports in which the RBC parameters were evaluated against other small molecules using aqueous or ethanolic extracts of *C. nutans* leaves in rats (Farsi *et al*. 2016; Mas’ ulun *et al*. 2021; Khoo *et al*. 2018), or methanolic extracts from *C. nutans* leaves in mice. Interestingly, other reports have demonstrated that daily repeated oral administration of the methanolic extract of *C. nutans* for 28 days in mice (Aliyu *et al*. 2020) and rats (Farsi *et al*. 2016) significantly increased MCH levels, suggesting that extracts from *C. nutans* leaves are capable of promoting haematopoiesis.

The estimated total WBC and differential WBC counts, that is, lymphocytes, monocytes, neutrophils, eosinophils and basophils in the present study were unchanged by administration of the CNBP extract at any acute or subacute dose. These results further support the view that the CNBP extract did not contain substances capable of inducing leucocytosis in the blood circulation, which is consistent with the findings reported by Farsi *et al*. (2016) and Zakaria *et al*. (2016). Similarly, there were no changes in the platelet count in the CNBP-treated rats compared to the control. This observation is consistent with the proposal that the CNBP extract used in this study does not induce thrombocytopenia or thrombocytosis.

## CONCLUSION

Acute toxicity tests revealed that a single oral dose of up to 3,000 mg/kg of CNBP extract did not cause any signs of toxicity during the subsequent 14 days, as assessed by physical, haematological and biochemical observations. However, in the subacute study, continuous administration of 500 mg/kg or higher doses of CNBP extract for 14 days caused a mild degree of hepatic toxicity, as characterised by elevated ALP levels. High doses (> 500 mg/kg body weight) of the extract induced mild histopathological alterations in both the acute and subacute groups. The CNBP extracts used in this study possess potential therapeutic activities such as diuresis, natriuresis, antihypertensive and cholesterol-lowering properties.

Precautions should be taken when administering the extracts at higher doses and longer durations, as potential adverse actions may become apparent. Based on the OECD-GL423 and GL407 guidelines, the LD_50_ of CNBP extract was higher than 3,000 mg/kg/day. However, in terms of the safety profile, the administration of CNBP extract should be lower than 500 mg/kg/day. This was based on the observation that the therapeutic effects of these extracts were apparent at a dose of 500 mg/kg/day. These findings provide new insights into the use of polysaccharides extracted from *C. nutans* leaves as a future treatment strategy for metabolic diseases.

## Figures and Tables

**Table 1:** Weekly physiological parameters of Sprague-Dawley rats administered with polysaccharides of *C. nutans* leaves over the 14 days study period in acute and subacute group. Data presented as mean ± SEM.

Parameter	Group	*n*	Day		

			0	7	14
Body weight (g)	C	3	244 ± 1	321 ± 2*	344 ± 3*
	A125	3	246 ± 2	319 ± 1*	337 ± 3*
	A250	3	254 ± 1	335 ± 3*	333 ± 3*
	A500	3	255 ± 0	312 ± 5*	333 ± 6*
	A1000	3	257 ± 1	314 ± 9*	310 ± 8*
	A2000	3	244 ± 3	310 ± 2*	333 ± 3*
	A3000	3	256 ± 2	333 ± 2*	332 ± 6*
	SA125	3	254 ± 1	324 ± 1*	335 ± 1*
	SA250	3	257 ± 2	296 ± 4*	336 ± 7*
	SA500	3	256 ± 1	291 ± 3*	315 ± 7*
	SA1000	3	255 ± 1	307 ± 1*	308 ± 6*
	SA2000	3	260 ± 3	334 ± 3*	344 ± 4*
	SA3000	3	261 ± 1	314 ± 4*	341 ± 2*

Water intake (mL)	C	3	45 ± 1	48 ± 1	48 ± 1
	A125	3	51 ± 0	54 ± 1	50 ± 1
	A250	3	46 ± 1	45 ± 1	42 ± 3
	A500	3	44 ± 1	45 ± 1	44 ± 2
	A1000	3	51 ± 2	51 ± 2	48 ± 6
	A2000	3	46 ± 1	50 ± 1	47 ± 1
	A3000	3	45 ± 2	44 ± 1	50 ± 1
	SA125	3	46 ± 1	49 ± 3	46 ± 2
	SA250	3	43 ± 2	43 ± 1	47 ± 3
	SA500	3	45 ± 2	44 ± 1	45 ± 3
	SA1000	3	48 ± 1	51 ± 1	48 ± 2
	SA2000	3	46 ± 1	47 ± 2	43 ± 3
	SA3000	3	48 ± 1	50 ± 3	46 ± 4

Urine output (mL)	C	3	21 ± 1	20 ± 1	18 ± 1
	A125	3	18 ± 3	20 ± 1	18 ± 1
	A250	3	19 ± 1	20 ± 2	18 ± 2
	A500	3	20 ± 3	22 ± 2	23 ± 2
	A1000	3	19 ± 2	25 ± 2	26 ± 2
	A2000	3	18 ± 2	25 ± 1	31 ± 1*,#
	A3000	3	18 ± 1	33 ± 1*	36 ± 1*,#
	SA125	3	19 ± 1	20 ± 1	23 ± 2
	SA250	3	19 ± 1	25 ± 2	26 ± 1
	SA500	3	18 ± 1	23 ± 2	24 ± 1
	SA1000	3	21 ± 1	30 ± 2*	32 ± 2*,#
	SA2000	3	19 ± 1	34 ± 3*	35 ± 1*,#
	SA3000	3	19 ± 1	41 ± 2*	43 ± 1*,#

Food intake (g)	C	3	36 ± 1	33 ± 1	37 ± 1
	A125	3	33 ± 3	36 ± 2	37 ± 2
	A250	3	33 ± 2	32 ± 1	29 ± 1
	A500	3	35 ± 2	35 ± 1	28 ± 1
	A1000	3	35 ± 2	34 ± 2	30 ± 2
	A2000	3	35 ± 1	35 ± 2	28 ± 2
	A3000	3	33 ± 1	32 ± 1	32 ± 1
	SA125	3	34 ± 2	35 ± 2	36 ± 2
	SA250	3	34 ± 2	34 ± 2	31 ± 4
	SA500	3	33 ± 3	30 ± 2	31 ± 1
	SA1000	3	36 ± 1	28 ± 1	29 ± 1
	SA2000	3	34 ± 2	28 ± 1	29 ± 2
	SA3000	3	35 ± 1	31 ± 2	32 ± 1

*Notes*:

* *p* < 0.05 of each group with respect to Day 0;

# *p* < 0.05 of all groups with respect to C on Day 14.

**Table 2:** Weekly non-invasive blood pressure parameters of Sprague-Dawley rats administered with polysaccharides of *C. nutans* leaves over the 14 days study period in acute and subacute group. Data presented as mean ± SEM.

Parameter	Group	*n*	Day		

			0	7	14
Systolic blood pressure (mmHg)	C	3	114 ± 1	115 ± 2	118 ± 2
	A125	3	117 ± 0	117 ± 3	116 ± 3
	A250	3	121 ± 1	118 ± 1	119 ± 1
	A500	3	109 ± 1	115 ± 2	109 ± 3
	A1000	3	119 ± 1	121 ± 3	112 ± 2
	A2000	3	110 ± 2	112 ± 0	107 ± 0#
	A3000	3	111 ± 1	107 ± 3	103 ± 4#
	SA125	3	115 ± 1	105 ± 2	110 ± 2
	SA250	3	115 ± 2	109 ± 2	108 ± 1
	SA500	3	114 ± 1	120 ± 2	105 ± 1#
	SA1000	3	111 ± 1	105 ± 3	102 ± 2#
	SA2000	3	116 ± 2	106 ± 3*	104 ± 2*,#
	SA3000	3	120 ± 2	105 ± 2*	102 ± 1*,#

Diastolic blood pressure (mmHg)	C	3	80 ± 1	75 ± 3	78 ± 0
	A125	3	73 ± 0	69 ± 0	75 ± 0
	A250	3	79 ± 2	71 ± 3	73 ± 3
	A500	3	77 ± 0	74 ± 1	73 ± 3
	A1000	3	80 ± 2	80 ± 3	75 ± 3
	A2000	3	76 ± 1	71 ± 1	71 ± 1
	A3000	3	74 ± 2	72 ± 1	69 ± 1
	SA125	3	76 ± 1	79 ± 4	76 ± 2
	SA250	3	82 ± 0	75 ± 1	75 ± 2
	SA500	3	78 ± 1	76 ± 1	69 ± 1
	SA1000	3	79 ± 1	76 ± 1	69 ± 1*
	SA2000	3	77 ± 2	71 ± 2	67 ± 3*
	SA3000	3	75 ± 1	66 ± 1	60 ± 1*

Mean arterial pressure (mmHg)	C	3	93 ± 1	95 ± 2	95 ± 0
	A125	3	91 ± 1	93 ± 0	94 ± 0
	A250	3	97 ± 1	96 ± 1	95 ± 2
	A500	3	92 ± 2	93 ± 3	92 ± 3
	A1000	3	99 ± 1	94 ± 2	89 ± 2*
	A2000	3	95 ± 1	91 ± 1	83 ± 1*
	A3000	3	94 ± 2	88 ± 3	83 ± 3*
	SA125	3	93 ± 1	94 ± 1	95 ± 1
	SA250	3	95 ± 2	94 ± 2	97 ± 1
	SA500	3	93 ± 1	92 ± 6	87 ± 2
	SA1000	3	96 ± 2	90 ± 1	86 ± 1*
	SA2000	3	93 ± 1	87 ± 2	84 ± 2*
	SA3000	3	92 ± 1	85 ± 2	77 ± 2*

Heart rate (BPM)	C	3	300 ± 1	298 ± 4	301 ± 2
	A125	3	304 ± 2	295 ± 1	295 ± 4
	A250	3	298 ± 2	297 ± 1	300 ± 2
	A500	3	296 ± 3	299 ± 2	292 ± 1
	A1000	3	303 ± 2	293 ± 3	286 ± 2*,#
	A2000	3	301 ± 2	285 ± 1*	286 ± 1*,#
	A3000	3	295 ± 1	283 ± 3*	284 ± 1*,#
	SA125	3	301 ± 1	304 ± 3	297 ± 1
	SA250	3	292 ± 3	295 ± 6	299 ± 1
	SA500	3	298 ± 2	293 ± 1	290 ± 2
	SA1000	3	302 ± 2	289 ± 1	281 ± 1*,#
	SA2000	3	307 ± 2	285 ± 1*	273 ± 2*,#
	SA3000	3	304 ± 1	288 ± 1*	267 ± 4*,#

*Notes*:

* *p* < 0.05 of each group with respect to Day 0;

# *p* < 0.05 of all groups with respect to C on Day 14.

**Table 3:** Haematology parameters; haemoglobin, RBC, PCV, MCV, MCH, MCHC and RDW of Sprague-Dawley rats administered with polysaccharides of *C. nutans* leaves at the end of day 14. Data presented as mean ± SEM.

Group	*n*	Parameter						

		Haemoglobin (g/dL)	RBC (× 10^12^/L)	PCV (%)	MCV (fL)	MCH (pg)	MCHC (g/dL)	RDW (%)
C	3	11.7 ± 0.2	4.14 ± 0.1	0.29 ± 0.03	63.0 ± 1.0	20.0 ± 1.0	31.6 ± 0.5	11.9 ± 0.3
A125	3	12.2 ± 0.5	4.19 ± 0.1	0.29 ± 0.04	62.0 ± 1.0	22.0 ± 1.0	32.7 ± 0.7	12.0 ± 0.2
A250	3	12.0 ± 0.2	4.76 ± 0.2	0.31 ± 0.02	65.0 ± 0.0	21.0 ± 1.0	32.3 ± 0.6	11.8 ± 0.3
A500	3	11.9 ± 0.4	5.59 ± 0.2	0.32 ± 0.02	64.0 ± 1.0	21.0 ± 0.0	32.1 ± 0.6	12.3 ± 0.7
A1000	3	12.3 ± 0.4	5.72 ± 1.0	0.32 ± 0.05	64.0 ± 1.0	20.0 ± 1.0	31.9 ± 0.2	11.8 ± 0.2
A2000	3	12.6 ± 0.8	5.93 ± 0.4	0.36 ± 0.04	65.0 ± 1.0	23.0 ± 1.0	31.7 ± 0.3	11.6 ± 0.2
A3000	3	12.4 ± 0.4	5.61 ± 0.6	0.32 ± 0.07	63.0 ± 1.0	22.0 ± 0.0	32.0 ± 0.2	11.7 ± 0.1
SA125	3	12.4 ± 0.2	4.40 ± 0.1	0.31 ± 0.02	61.0 ± 1.0	25.0 ± 2.0	32.7 ± 0.7	12.0 ± 0.2
SA250	3	12.3 ± 0.4	4.53 ± 0.2	0.28 ± 0.01	62.0 ± 1.0	20.0 ± 0.0	33.1 ± 0.1	11.9 ± 0.2
SA500	3	13.2 ± 0.6	5.59 ± 0.3	0.36 ± 0.04	62.0 ± 1.0	20.0 ± 1.0	32.5 ± 0.6	11.7 ± 0.1
SA1000	3	13.6 ± 0.1	5.52 ± 0.6	0.29 ± 0.02	61.0 ± 1.0	21.0 ± 0.0	33.3 ± 0.6	11.5 ± 0.2
SA2000	3	13.1 ± 0.5	6.04 ± 0.1	0.31 ± 0.01	61.0 ± 1.0	23.0 ± 1.0	31.8 ± 0.4	11.9 ± 0.2
SA3000	3	13.5 ± 0.2	6.06 ± 0.1	0.32 ± 0.03	61.0 ± 1.0	22.0 ± 2.0	32.6 ± 0.5	12.1 ± 0.1

*Notes*: RBC = red blood cell, PCV = packed cell volume, MCV = mean corpuscular volume, MCH = mean corpuscular haemoglobin, MCHC = mean corpuscular haemoglobin concentration, RDW = red cell distribution width

**Table 4:** Haematology parameters; WBC, lymphocytes, monocytes, neutrophils, basophils, eosinophils and platelets count of Sprague-Dawley rats administered with polysaccharides of *C. nutans* leaves at the end of day 14. Data presented as mean ± SEM.

Group	*n*	Parameter						

		WBC (×10^9^/L)	Lymphocytes (%)	Monocytes (%)	Neutrophils (%)	Eosinophils (%)	Basophils (%)	Platelets (×10^5^/μL)
C	3	3.93 ± 0.36	71.3 ± 1.2	1.3 ± 0.8	23.3 ± 1.2	2.7 ± 0.8	0.3 ± 0.4	2.50 ± 0.18
A125	3	4.23 ± 0.20	76.3 ± 0.8	1.7 ± 0.4	22.7 ± 2.4	3.3 ± 0.4	0.7 ± 0.4	2.86 ± 0.35
A250	3	3.50 ± 0.86	81.0 ± 2.4	2.0 ± 0.0	22.0 ± 2.9	3.0 ± 1.2	1.0 ± 0.4	3.14 ± 0.98
A500	3	4.43 ± 0.69	73.3 ± 1.2	1.3 ± 0.4	21.3 ± 1.6	3.7 ± 0.4	1.0 ± 0.4	3.72 ± 0.88
A1000	3	4.63 ± 0.65	80.0 ± 2.4	1.7 ± 0.8	20.7 ± 2.4	2.7 ± 0.1	1.0 ± 0.8	3.42 ± 0.53
A2000	3	4.40 ± 0.09	73.0 ± 2.4	1.3 ± 0.0	22.0 ± 2.4	4.0 ± 0.8	1.3 ± 0.0	3.85 ± 0.69
A3000	3	3.84 ± 0.00	76.3 ± 0.8	1.3 ± 0.4	26.0 ± 0.4	3.0 ± 0.8	2.0 ± 0.8	3.73 ± 0.51
SA125	3	4.11 ± 0.30	77.3 ± 0.9	1.0 ± 0.6	27.0 ± 3.1	2.7 ± 0.9	0.7 ± 0.3	3.10 ± 0.95
SA250	3	3.80 ± 0.27	73.7 ± 0.3	1.7 ± 0.3	22.0 ± 0.6	3.0 ± 0.6	1.3 ± 0.3	2.05 ± 0.83
SA500	3	4.27 ± 0.24	71.7 ± 1.8	2.0 ± 0.6	24.3 ± 2.7	2.3 ± 0.9	1.7 ± 0.3	2.69 ± 0.74
SA1000	3	5.10 ± 0.51	75.0 ± 3.2	1.7 ± 0.3	24.7 ± 1.9	3.3 ± 0.9	1.0 ± 0.6	2.28 ± 0.91
SA2000	3	4.18 ± 0.42	75.7 ± 2.7	1.3 ± 0.3	28.0 ± 2.6	3.0 ± 1.2	0.7 ± 0.3	3.88 ± 0.52
SA3000	3	4.06 ± 0.44	76.0 ± 3.8	2.0 ± 0.6	27.3 ± 3.2	4.0 ± 0.6	1.3 ± 0.3	3.27 ± 0.76

*Note*: WBC = white blood cell

**Table 5:** Blood and urine electrolyte parameters; urine sodium, plasma sodium, urine potassium and plasma potassium of Sprague-Dawley rats administered with polysaccharides of *C. nutans* leaves at the end of day 14. Data presented as mean ± SEM.

Group	*n*	Parameter				

		Urine sodium (mmol/L)	Plasma sodium (mmol/L)	Fractional excretion of sodium (%)	Urine potassium (mmol/L)	Plasma potassium (mmol/L)
C	3	47 ± 1	140 ± 4	0.15 ± 0.16	140 ± 3	6.2 ± 0.2
A125	3	44 ± 1	135 ± 7	0.16 ± 0.07	143 ± 0	5.8 ± 0.1
A250	3	61 ± 1	139 ± 2	0.18 ± 0.01	144 ± 0	5.3 ± 0.1
A500	3	79 ± 1#	134 ± 4	0.25 ± 0.07#	145 ± 1	5.6 ± 0.1
A1000	3	82 ± 3#	141 ± 2	0.25 ± 0.02#	139 ± 0	5.8 ± 0.2
A2000	3	82 ± 2#	137 ± 3	0.24 ± 0.01#	144 ± 1	6.1 ± 0.1
A3000	3	80 ± 2#	138 ± 7	0.28 ± 0.01#	140 ± 3	6.0 ± 0.4
SA125	3	45 ± 2	143 ± 1	0.19 ± 0.03	142 ± 2	5.5 ± 0.4
SA250	3	77 ± 2#	134 ± 2	0.17 ± 0.01	141 ± 1	6.0 ± 0.3
SA500	3	84 ± 4#	132 ± 7	0.25 ± 0.01#	140 ± 1	5.6 ± 0.2
SA1000	3	87 ± 3#	136 ± 6	0.26 ± 0.02#	141 ± 2	5.7 ± 0.2
SA2000	3	88 ± 2#	141 ± 2	0.26 ± 0.01#	139 ± 1	5.6 ± 0.2
SA3000	3	90 ± 2#	143 ± 7	0.22 ± 0.01#	145 ± 1	6.0 ± 0.2

*Note*:

# *P* < 0.05 of all groups with respect to C on Day 14.

**Table 6:** Renal functional parameters; urine creatinine, plasma creatinine, plasma urea, plasma chloride and fasting blood glucose index of Sprague-Dawley rats administered with polysaccharides of *C. nutans* leaves at the end of day 14. Data presented as mean ± SEM.

Group	*n*	Parameter					

		Urine creatinine (mmol/L)	Plasma creatinine (mmol/L)	Creatinine clearance (mL/min/mol/kg)	Plasma urea (mmol/L)	Plasma chloride (mmol/L)	Fasting blood glucose index (mmol/L)
C	3	4.54 ± 0.02	20 ± 1	2.73 ± 0.12	4.4 ± 0.1	104 ± 1	5.4 ± 0.2
A125	3	4.82 ± 0.01	21 ± 0	2.98 ± 0.03	4.5 ± 0.2	102 ± 2	5.3 ± 0.1
A250	3	5.11 ± 0.27	22 ± 1	2.88 ± 0.13	4.6 ± 0.1	101 ± 1	5.3 ± 0.2
A500	3	5.38 ± 0.10#	24 ± 2	3.63 ± 0.40#	4.9 ± 0.4	101 ± 1	5.2 ± 0.2
A1000	3	5.57 ± 0.18#	23 ± 3	4.46 ± 0.45#	5.4 ± 0.1	99 ± 1	5.5 ± 0.1
A2000	3	5.79 ± 0.09#	25 ± 2	5.08 ± 0.46#	5.2 ± 0.2	101 ± 1	5.3 ± 0.2
A3000	3	5.73 ± 0.05#	24 ± 2	6.02 ± 0.18#	4.1 ± 0.1	100 ± 1	5.6 ± 0.2
SA125	3	4.85 ± 0.06	23 ± 2	3.72 ± 0.30	5.3 ± 0.2	104 ± 2	5.1 ± 0.1
SA250	3	5.93 ± 0.15#	18 ± 1	5.89 ± 0.41#,¥	4.0 ± 0.2	99 ± 1	6.1 ± 0.3
SA500	3	5.98 ± 0.34#	21 ± 1	4.75 ± 0.24#	5.2 ± 0.5	100 ± 1	5.3 ± 0.2
SA1000	3	5.93 ± 0.17#	25 ± 2	5.39 ± 0.25#	5.5 ± 0.2	101 ± 1	5.0 ± 0.1
SA2000	3	5.95 ± 0.17#	24 ± 2	6.14 ± 0.41#	4.6 ± 0.2	103 ± 2	5.7 ± 0.3
SA3000	3	5.97 ± 0.19#	21 ± 1	8.45 ± 0.31#,¥	4.8 ± 0.5	104 ± 2	5.4 ± 0.1

*Notes*: *P* < 0.05 of all groups with respect to C on Day 14;

¥ *P* < 0.05 of A group versus SA group with similar dose on Day 14.

**Table 7:** Liver functional parameters; total protein, albumin, globulin and albumin/globulin ratio of Sprague-Dawley rats administered with polysaccharides of *C. nutans* leaves at the end of day 14. Data presented as mean ± SEM.

Group	*n*	Parameter			

		Total protein (g/L)	Albumin (g/L)	Globulin (g/L)	Albumin/Globulin ratio
C	3	44 ± 2	35 ± 2	10 ± 1	2.7 ± 0.2
A125	3	43 ± 1	35 ± 2	13 ± 1	2.6 ± 0.2
A250	3	47 ± 3	36 ± 2	11 ± 1	3.2 ± 0.1
A500	3	45 ± 1	33 ± 0	12 ± 1	2.9 ± 0.2
A1000	3	47 ± 2	35 ± 2	12 ± 0	3.0 ± 0.1
A2000	3	45 ± 1	35 ± 2	11 ± 2	3.3 ± 0.4
A3000	3	51 ± 0	38 ± 1	13 ± 1	3.2 ± 0.3
SA125	3	49 ± 3	36 ± 2	14 ± 2	2.7 ± 0.3
SA250	3	48 ± 1	34 ± 1	14 ± 0	2.4 ± 0.1
SA500	3	47 ± 2	34 ± 2	14 ± 1	2.5 ± 0.3
SA1000	3	47 ± 1	35 ± 2	12 ± 1	2.9 ± 0.5
SA2000	3	46 ± 1	35 ± 2	14 ± 1	2.7 ± 0.1
SA3000	3	48 ± 2	37 ± 1	13 ± 1	2.9 ± 0.1

**Table 8:** Liver functional parameters; total bilirubin, alkaline phosphatase, gamma-glutamyl transferase, aspartate aminotransferase and alanine aminotransferase of Sprague-Dawley rats administered with polysaccharides of *C. nutans* leaves at the end of day 14. Data presented as mean ± SEM.

Group	*n*	Parameter				

		Total bilirubin (μmol/L)	Alkaline phosphatase (U/L)	Gamma-glutamyl transferase (U/L)	Aspartate aminotransferase (U/L)	Alanine aminotransferase (U/L)
C	3	<2 ± 0	163 ± 9	<3.0 ± 0.0	115 ± 1	37 ± 2
A125	3	<2 ± 0	175 ± 2	<3.0 ± 0.0	121 ± 7	40 ± 0
A250	3	<2 ± 0	169 ± 7	<3.0 ± 0.0	107 ± 6	40 ± 4
A500	3	<2 ± 0	188 ± 4	3.3 ± 0.3	126 ± 4	58 ± 1
A1000	3	<2 ± 0	184 ± 4	3.7 ± 0.3	142 ± 6	59 ± 4
A2000	3	<2 ± 0	180 ± 9	<3.0 ± 0.0	126 ± 8	53 ± 9
A3000	3	<2 ± 0	173 ± 3	3.3 ± 0.0	114 ± 4	40 ± 4
SA125	3	<2 ± 0	174 ± 4	<3.0 ± 0.0	119 ± 1	37 ± 4
SA250	3	<2 ± 0	185 ± 5	3.3 ± 0.3	121 ± 4	39 ± 3
SA500	3	<2 ± 0	222 ± 49#, ¥	3.3 ± 0.3	118 ± 10	55 ± 2
SA1000	3	<2 ± 0	223 ± 29#, ¥	<3.0 ± 0.0	120 ± 8	56 ± 3
SA2000	3	<2 ± 0	238 ± 33#, ¥	3.3 ± 0.3	114 ± 7	42 ± 3
SA3000	3	<2 ± 0	252 ± 18#, ¥	<3.0 ± 0.0	113 ± 2	38 ± 3

*Notes*:

# *P* < 0.05 of all groups with respect to C on Day 14;

¥ *P* < 0.05 of A group versus SA group with similar dose on day 14.

**Table 9:** Lipid pro le; total cholesterol, high-density lipoprotein, low-density lipoprotein, non-high-density lipoprotein cholesterol, triglycerides and total cholesterol/high-density lipoprotein ratio of Sprague-Dawley rats administered with polysaccharides of *C. nutans* leaves at the end of day 14. Data presented as mean ± SEM.

Group	*n*	Parameter					

		Total cholesterol (mmol/L)	HDL (mmol/L)	LDL (mmol/L)	Non-HDL (mmol/L)	Triglycerides (mmol/L)	Total cholesterol/HDL ratio
C	3	1.9 ± 0.1	0.83 ± 0.03	0.81 ± 0.03	1.07 ± 0.05	0.27 ± 0.01	2.3 ± 0.0
A125	3	1.8 ± 0.1	0.90 ± 0.01	0.80 ± 0.03	0.94 ± 0.04	0.28 ± 0.01	2.0 ± 0.1
A250	3	1.8 ± 0.1	0.93 ± 0.01	0.87 ± 0.00	0.87 ± 0.07	0.27 ± 0.05	1.9 ± 0.1
A500	3	1.7 ± 0.1	0.96 ± 0.09	0.80 ± 0.04	0.78 ± 0.03#	0.28 ± 0.01	1.8 ± 0.1
A1000	3	1.6 ± 0.3	0.99 ± 0.03	0.81 ± 0.02	0.72 ± 0.03#	0.30 ± 0.02	1.6 ± 0.3
A2000	3	1.7 ± 0.1	0.95 ± 0.04	0.83 ± 0.01	0.75 ± 0.08#	0.29 ± 0.02	1.8 ± 0.1
A3000	3	1.8 ± 0.2	0.99 ± 0.05	0.80 ± 0.03	0.78 ± 0.01#	0.27 ± 0.00	1.8 ± 0.1
SA125	3	1.8 ± 0.1	0.89 ± 0.03	0.79 ± 0.02	0.95 ± 0.10	0.29 ± 0.03	2.1 ± 0.1
SA250	3	1.8 ± 0.2	0.98 ± 0.03	0.55 ± 0.01#, ¥	0.79 ± 0.15#	0.34 ± 0.03	1.8 ± 0.1
SA500	3	1.8 ± 0.2	0.97 ± 0.02	0.52 ± 0.04#, ¥	0.79 ± 0.19#	0.31 ± 0.01	1.8 ± 0.2
SA1000	3	1.5 ± 0.0	0.95 ± 0.02	0.54 ± 0.02#, ¥	0.55 ± 0.02#, ¥	0.33 ± 0.03	1.6 ± 0.0
SA2000	3	1.8 ± 0.1	0.97 ± 0.01	0.55 ± 0.02#, ¥	0.59 ± 0.01#, ¥	0.32 ± 0.02	1.8 ± 0.1
SA3000	3	1.6 ± 0.2	0.98 ± 0.02	0.52 ± 0.03#, ¥	0.58 ± 0.17#, ¥	0.33 ± 0.03	1.6 ± 0.2

*Notes*:

# *P* < 0.05 of all groups with respect to C on Day 14;

¥ *P* < 0.05 of A group versus SA group with similar dose on day 14.

**Table 10:** Relative organ weight of Sprague-Dawley rats administered with polysaccharides of *C. nutans* leaves at the end of day 14. Data presented as mean ± SEM.

Parameter	Group	*n*	Liver	Kidney
Relative organ weight (g)	C	3	3.01 ± 0.47	0.31 ± 0.03
	A125	3	2.96 ± 0.18	0.35 ± 0.02
	A250	3	3.20 ± 0.49	0.37 ± 0.04
	A500	3	3.22 ± 0.57	0.36 ± 0.06
	A1000	3	3.18 ± 0.47	0.38 ± 0.12
	A2000	3	3.26 ± 0.20	0.41 ± 0.02
	A3000	3	3.28 ± 0.79	0.38 ± 0.03
	SA125	3	3.19 ± 0.38	0.34 ± 0.02
	SA250	3	3.17 ± 0.17	0.35 ± 0.03
	SA500	3	3.07 ± 0.09	0.39 ± 0.09
	SA1000	3	3.04 ± 0.35	0.33 ± 0.05
	SA2000	3	3.18 ± 0.42	0.35 ± 0.03
	SA3000	3	3.32 ± 0.90	0.37 ± 0.03

## Data Availability

Data is available from the corresponding authors on request.
